# 3D X-ray Micro-CT Analysis of Rebar Corrosion in Reinforced Concrete Subjected to a Chloride-Induced Environment

**DOI:** 10.3390/molecules27010192

**Published:** 2021-12-29

**Authors:** Łukasz Skarżyński, Katarzyna Kibort, Aleksandra Małachowska

**Affiliations:** 1 Department of Building Structures and Material Engineering, Faculty of Civil and Environmental Engineering, Gdansk University of Technology, G. Narutowicza St. 11/12, 80-233 Gdansk, Poland; 2 Department of Process Engineering and Chemical Technology, Faculty of Chemistry, Gdansk University of Technology, G. Narutowicza St. 11/12, 80-233 Gdansk, Poland; katkibor@student.pg.edu.pl (K.K.); aleksandra.malachowska@pg.edu.pl (A.M.)

**Keywords:** bond strength, chloride-induced corrosion, concrete cover, pull-out test, reinforced concrete, X-ray micro-CT, 3D reconstruction

## Abstract

The paper presents experimental investigations of the concrete covers’ protective ability to counteract rebar corrosion in reinforced concrete cubes. The concrete sample was subjected to a chloride-induced environment to get corroded and combined with an un-corroded sample. The chloride-accelerated technique can induce a high degree of corrosion within a controlled time. Moreover, detailed and thorough experimental measurements and analyses of reinforcement loss due to corrosion and its influence on concrete microstructure, were studied through 3D X-ray micro-computed tomography. The rebar outside the concrete was heavily corroded due to the chloride-accelerated test, whereas, only local surface corrosion products appeared inside the concrete. It turned out that the concrete cover showed protective ability to counteract the reinforcing-steel corrosion mechanism despite the accelerated corrosion environment. Moreover, the bond strength between the reinforcement rebar and concrete was not visibly affected since the failure force in the pull-out test and failure mechanisms, observed by 3D X-ray micro-CT, were similar for corroded and un-corroded samples. The failure occurred due to radial cracks with a maximum width equal to approximately 0.25 mm.

## 1. Introduction

The latest reports have shown significant economic impact due to the negative influence of corrosion. Thus, some countries are forced to spend money on repairing and maintaining steel and RC structures [1,2]. Corrosion of steel reinforcement is currently one of the most critical problems in preserving concrete structures. Corrosion of reinforcement leads to the weakening of the bond strength, resulting in the deterioration of the structural integrity. As a result of corrosion, the initial cross-section of the reinforcement is reduced, which weakens the mechanical properties of the steel bar.

Moreover, tensile stresses in the concrete cover, caused by the oxides on the corroded steel, lead to loss of the bond mechanism. Simultaneously, the high alkalinity of concrete (pH ~ 13.0) protects the steel due to a passive zone of ferric oxides. Thus, steel reinforcement is protected by a passive layer on its surface and concrete cover. Significantly, the uncracked concrete cover is a physical barrier for corrosive factors delaying their diffusion through the concrete pores towards the reinforcement. Chlorides enter the concrete pore network by diffusion or capillary suction or combining the mechanisms as mentioned above [3]. Before chloride ions reach the steel reinforcement, there is an initiation period. During this period, water and chloride ions reach the appropriate level necessary to start the corrosion process [4].

This process can take more than 10 or 15 years until the destructive environmental influence reaches the reinforcement. At the same time, the alkalinity of the concrete decreases (pH reduces below nine), and the thin protective layer on the reinforcement starts to depassivate. Then, oxidation occurs, and a gradual increase of rust on the steel reinforcement occurs. For the past few decades, many experimental and theoretical works have been dedicated to investigating the corrosion of steel reinforcement, however usually at the structural level (macro-scale). Most recent studies utilize the typical application of X-ray computed tomography and other innovative techniques to visualize phenomena such as steel corrosion and its progression in real-time, at the micrometer and even nanometer scale. In many studies, experimental work is performed on crack-induced concrete material to accelerate corrosion. In the study of Savija et al. [5], several experiments were performed to analyze different aspects of cracking of the protective cover due to reinforcing-steel corrosion. Tests included an accelerated corrosion test in saturated calcium hydroxide (Ca (OH)_2_) solution. Non-destructive monitoring of the corrosion process in reinforced specimens was performed through X-ray CT scans. In addition, an innovative nanoindentation technique was used. The result indicated that some mechanical properties of rust (e.g., Young’s modulus) are highly dependent on the level of confinement provided by the surrounding cement paste to the rust layer. Cheng et al. [6] presented a novel non-contact capacitive transducer (CT) as an alternative hardware sensor that directly determined the rust layer thickness. Corrosion simulations were conducted using two different corrosion-volume expansion ratios on rebar of various diameters, and the direct determination of the rust layer thickness was identified. The method was more sensitive for rebar with a larger diameter and higher volume expansion ratio. The results demonstrated that the developed capacitive sensor could be highly effective for determining the thickness of the rust layer of various rebar. Shi et al. [7] determined the main corrosion products and their distribution at the steel—concrete interface and the pattern of corrosion-induced cracks. The experiment was based on the accelerated corrosion of steel in cubic concrete with 3.5% NaCl solution using CT-scans and an Environmental Scanning Electron Microscope (ESEM). Results revealed cracks in the rust layer of the steel and a thin layer of corrosion appeared in the steel—concrete interface. The rust layer of low-alloy steel can be assumed to be the loose outer layer and could penetrate the nearby cement paste, and the inner layer that is compacted and enriched with Cr. With the acoustic emission (AE) technique and validation via X-ray CT, Steen et al. [8] have proven that AE can accurately identify damage due to corrosion in reinforced concrete. Accelerated corrosion (5% NaCl solution) of rebar in concrete was utilized. The change in AE energy allowed us to determine the cracking moment that resulted in clear differentiation between cracked and uncracked samples. Xi and Yang [9] analyzed and discussed the corrosion-products expansion in crack-induced concrete. The experimental study used chloride-accelerated corrosion and monitored the corrosion progression of a corner-located steel bar in concrete, investigated by X-ray computed tomography. The test accelerated corrosion of a single rebar, in diluted NaCl solution, and investigated it via X-CT, SEM & EDS. The combination of a wetting and drying cyclic corrosive environment and the X-CT scanning, supported with SEM and EDS, provided an innovative approach to the non-destructive investigation of the corrosion process, rust distribution and corrosion-induced concrete cracking in the reinforced concrete structures. Wang et al. [10] studied steel corrosion and its distribution and corrosion-induced crack progression of magnesium oxychloride cement concrete (MOCC). Experimental work has accelerated steel corrosion in cubic concrete and saline soil. The investigation was made with SEM. X-ray 3D CT scans were used to monitor the accumulating corrosion products. It resulted in corrosion gradually spreading into the cement matrix. A conclusion was made regarding the relationship between the volume increase in corrosion-induced cracks and the increase in crack width. The progression of corrosion-induced cracks started from the concrete’s external surface and increased gradually with steel corrosion. Filho et al. [11] determined the amount of reinforcement lost to corrosion through X-ray tomography on samples with induced cracking. The experimental work used an accelerated corrosion test with 3.5% NaCl solution, future SEM, and CT scan evaluation. The tomography scans allowed for scanning without removing the concrete cover to determine the actual damage present in the samples. The results were consistent with estimations by other electrical and electrochemical methods mentioned herein. Recently, tests concerning corrosion of steel reinforcement and its influence on compressive behaviour of concrete [12], shear strength of RC beams [13], and damage characteristics of steel fiber concrete [14] were carried out.

## 2. Significance of the Research

The main goal of this research was to better understand the protective ability of the concrete cover to counteract the reinforcing-steel corrosion mechanism that negatively affects bond strength. This knowledge is essential for enhancing the lifespan of reinforced concrete members and structures. Bond strength is the maximum bond stress, mainly resulting from friction between reinforcement rebars and the concrete, which can easily be regarded as shear stress over the bar’s surface or the interlocking mechanism, along with the reinforcing bar interface with the surrounding concrete. The bond between the concrete and reinforcement involves three effective mechanisms: adhesion, friction between concrete and steel surface, and the bearing of reinforcement ribs against concrete. To study and quantify the consequences of corrosion, laboratory methods have been developed to simulate and accelerate the natural process in reinforced concrete samples. The research procedure performed a chloride-accelerated corrosion test combined with X-ray micro-CT observations carried out at different intervals. Finally, the two main novel objectives of the study were:

1. Detailed and thorough 3D experimental measurements and analyses of reinforcement loss due to corrosion and its influence on concrete microstructure using X-ray micro-computed tomography
2. 3D X-ray micro-computed 3D investigations of fractures in reinforced concrete samples subjected to pull-out tests.

## 3. Sample Preparation

Table 1 presents the proportions used in the concrete recipe. The sand point was equal to 41%, whereas the water to cement ratio was assumed to be w/c = 0.50. Before the molding procedure, the fresh concrete was tested using the Vebe slump test, Vebe time test, and air content pressure test [15]. Results of the new concrete mix analyses are depicted in Table 2. Acceptable concrete mix workability and air content were proved. Both abovementioned parameters have little influence on the final properties of the hardened concrete, such as strength, permeability, and durability.

**Figure 1:**
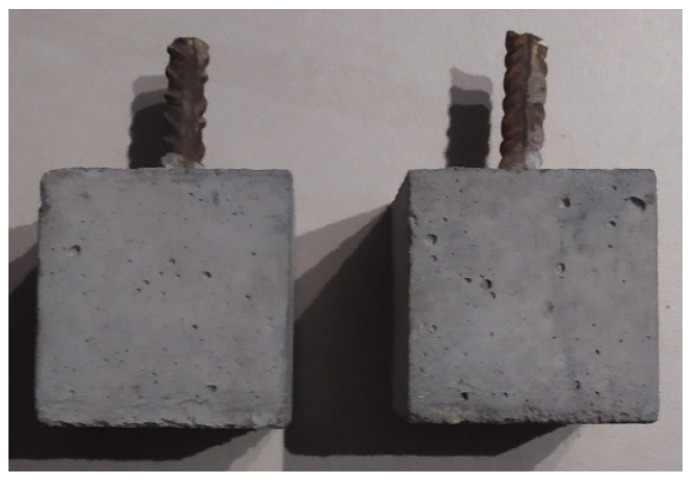
Reinforced concrete cubes for experimental procedure.

Finally, two cubic concrete samples with dimensions of 70 × 70 × 70 mm reinforced with a centrally located steel bar with a diameter of 12 mm were prepared; thus, the concrete cover was equal to 29 mm (Figure 1). The reinforcement ratio value was ρ = 2.1%. The tensile strength of the steel wad was *fys* = 650 MPa, and the modulus of elasticity of steel was *Es* = 200 GPa. For the first seven days, blocks were stored in a climatic chamber at a temperature of about 200C and humidity 95% to avoid surface evaporation and autogenous shrinkage. Relatively small dimensions for the reinforced concrete specimens were used to enable the entire sample to be scanned in the micro-CT system.

Mechanical tests on the hardened concrete (on the 28th day after concreting) were conducted on six cubic specimens with the dimensions of 15 × 15 × 15 cm. The average uniaxial compressive strength [16] was fc = 38.60 MPa with a standard deviation of 1.94 MPa; while the average splitting strength [17] was fct = 2.96 MPa with the standard deviation of 0.25 MPa. The Young’s modulus was E = 32.4 GPa with the standard deviation of 1.89 GPa, and the Poisson’s ratio was υ = 0.21 with a standard deviation of 0.02. Both parameters were tested on three cylinder specimens (15 × 30 cm). The average flexural strength [18] tested on three concrete beams (15 × 15 × 60 cm), was fcf = 3.60 MPa with a standard deviation of 0.2 MPa.

## 4. Initial X-ray Micro-CT Scanning

**Figure 2:**
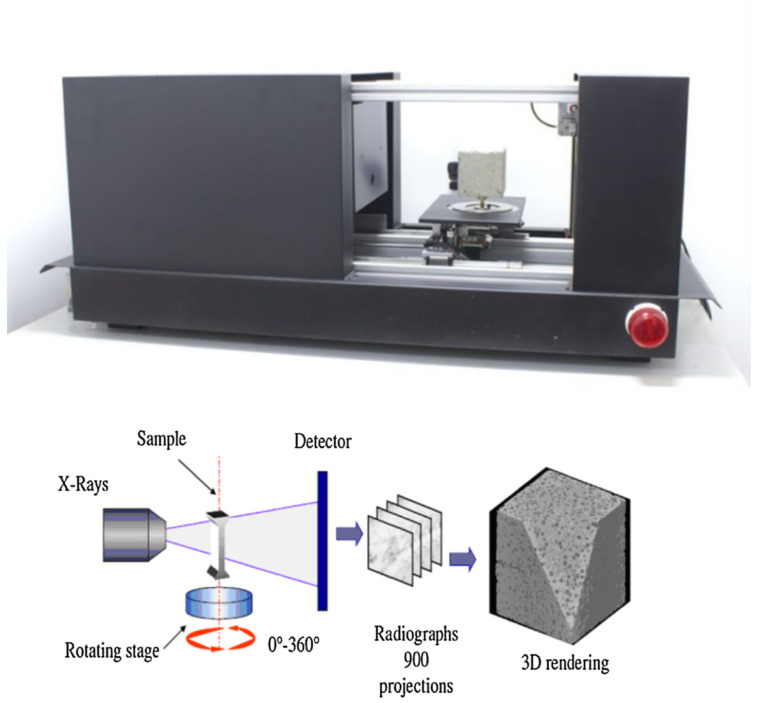
X-ray Computed Tomography SkyScan 1173 and the operation principles of X-ray micro-CT.

Micro-computed tomography is a non-destructive 3D imaging technique using X-ray to see the inside of objects. The micro-CT scanner makes a series of 2D planar X-ray images and reconstructs the data into 2D cross-sectional slices that are further processed into 3D models. Thus, the volumetric information about changes in the internal micro/mesostructure may be obtained. There are many examples of micro-CT systems for concrete members (e.g., [19,20,21,22,23,24,25,26]). Our tomography system SkyScan 1173 has already been used successfully to observe the evolution of a concrete fracture process during three-point bending in plain concrete [27]. Other investigations included tension splitting in plain concrete [28], uniaxial compression in plain concrete [29], compressive fatigue in plain concrete [30], three-point bending in plain concrete under constant scanning [31], and steel or basalt fibrous concrete subjected to wedge splitting [32,33]. An X-ray micro-CT SkyScan 1173 scanner with 0.2 mm brass filter was used to investigate the 3D material microstructure and fracture properties. The X-ray source voltage and current were 130 keV and 61 µA, respectively. The voxel size of the X-ray micro-CT was 39.68 microns, whereas the shutter speed was 5000 ms. The sample was scanned at 360 degrees with a single rotation step of 0.4 degrees. The experimental procedure started from an initial micro-CT scan of non-cracked samples to gather all necessary information concerning initial porosity and material microstructure. The testing station used for micro-CT experiments is presented in Figure 2.

**Figure 3:**
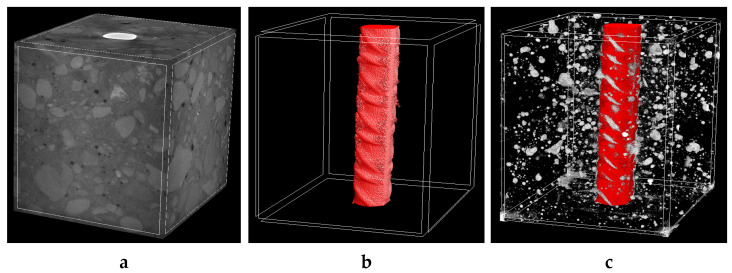
The 3D visualization of reinforced concrete sample no. 1: (**a**) entire sample, (**b**) steel reinforcement bar and (**c**) steel reinforcement bar with air void distribution.

**Figure 4:**
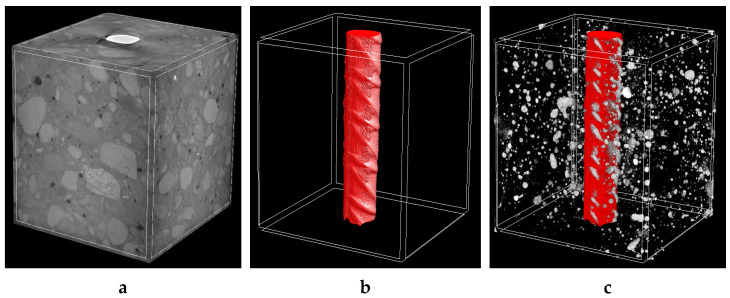
The 3D visualization of reinforced concrete sample no. 2: (**a**) entire sample, (**b**) steel reinforcement bar and (**c**) steel reinforcement bar with air void distribution.

Figure 3 and Figure 4 show the 3D visualization of the reinforced concrete sample, a reinforcement, and air voids distribution in non-cracked reinforced concrete measured by X-ray micro-CT. Air voids were separated using a threshold value between 0–60 (within the whole 0–255 scale). They were treated in two ways, i.e., as open pores that cross the boundaries of the VOI (Volume of Interest) or closed pores that are entirely embedded in the VOI. Based on that assumption, the air volume in the non-cracked reinforced concrete specimens ranged from 2.75% to 2.81% of total air volume. In contrast, closed porosity varied from 2.39% to 2.49% and open porosity varied from 0.55% to 0.61%.

## 5. Corrosion Test

**Figure 5:**
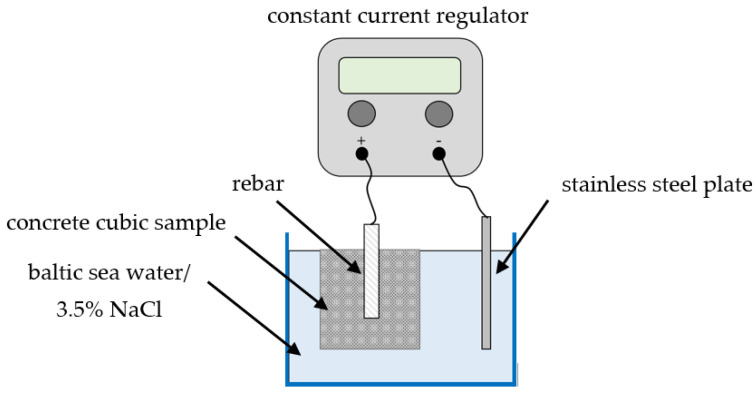
Schematic illustration of the experimental set-up for corrosion stimulation.

To study and quantify the consequences of corrosion, laboratory methods have been developed to simulate and accelerate the natural process. The research procedure consisted of performing a chloride-accelerated corrosion test. This technique can induce a high degree of corrosion within a controlled time. The tests were carried out by immersing a cubic concrete sample (70 × 70 × 70 mm) with rebar, initially in a reservoir with Baltic Seawater, in order to simulate the marine environment. After seven days, no changes were observed, and due to that, to intensify the corrosion process, we increased the salinity to 3.5% by adding NaCl to the solution. Direct electric current was applied to the reinforcing steel bars through the power supply to accelerate the corrosion’s electrochemical reaction. The voltage value was set at 2.5 V. The positive-constant-current regulator electrode was connected to the rebar and the negative electrode to the stainless steel plate, which was also immersed in NaCl solution. In this way, an electric circuit was generated. The reinforcing steel bars acted as the anode of the circuit, the stainless steel plate acted as a cathode, and the saltwater was the electrolyte, which allowed ions to flow into the circuit [6,7,10]. Figure 5 shows a schematic illustration of the experimental setup for the corrosion simulation. The main advantage of this method is the ability to control the corrosion rate, which usually varies due to changes in resistivity, oxygen concentration, and temperature. However, there are many uncertainties since many factors influence the rate of chloride penetration into concrete, such as porosity and cracks, temperature, moisture, and salinity of corrosive environment.

**Figure 6:**
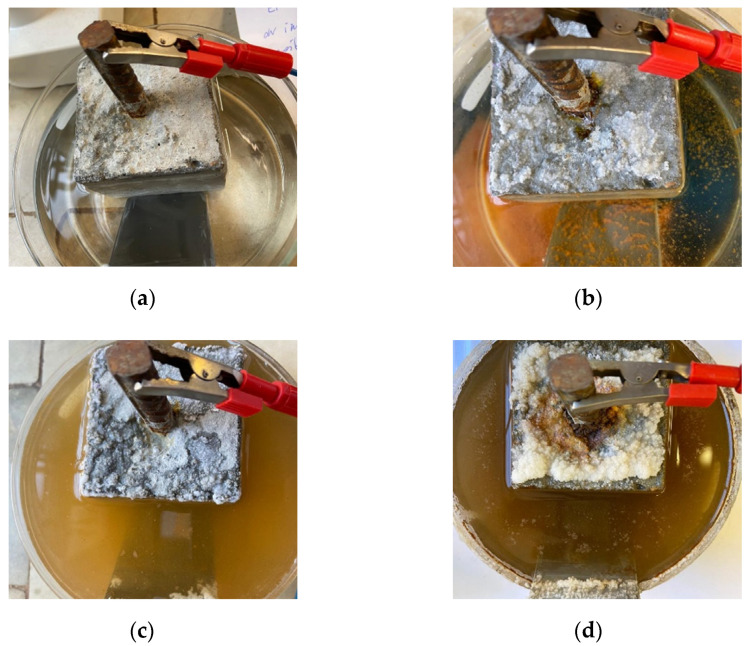
Corrosion acceleration experiment of single rebar: (**a**) first day of the experiment, immersing a concrete cube with rebar in a NaCl solution, (**b**) 14th day of the experiment, noticeable first corrosion changes within the rebar, (**c**) 42nd day of the experiment, increased rebar corrosion, beginning of salt crystallization on the concrete cube, (**d**) 70th day of the experiment, strong corrosion of the rebar radiating to the concrete cube, extensive salt crystallization.

**Figure 7:**
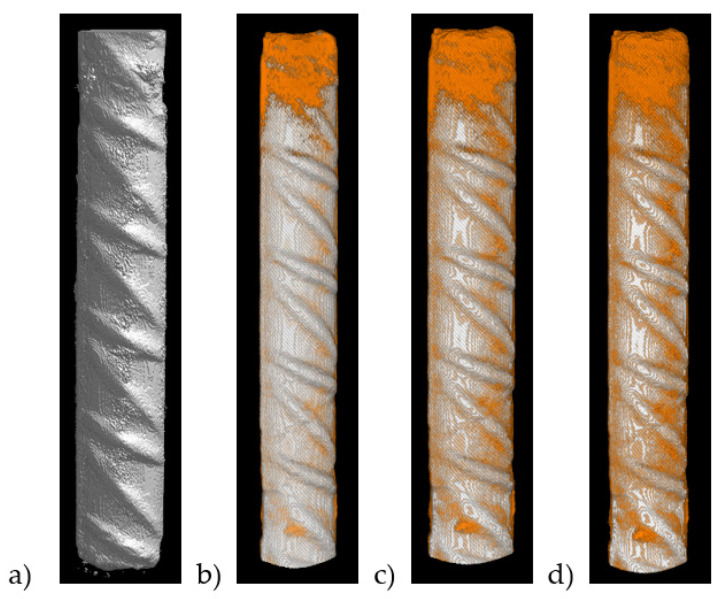
The 3D X-ray micro-CT visualization of steel rebar: (**a**) before corrosion acceleration experiment, (**b**) 14th day of the experiment, noticeable first corrosion changes within the rebar, (**c**) 42nd day of the experiment and, (**d**) 70th day of the experiment.

Figure 6 shows changes observed after a single rebar corrosion acceleration experiment for several days. After 14 days of the experiment, noticeable first corrosion changes within the rebar were noticed. Next, after 42 days, increased rebar corrosion and the beginning of salt crystallization on the concrete cube appeared. Furthermore, after 70 days of the experiment, strong corrosion of rebar combined with extensive salt crystallization could be observed. Simultaneously, X-ray micro-CT scanning tests were carried out while the evolution of corrosion was investigated in 3D (Figure 7).

The ribbing of the reinforcing bar is distinguished. After 14 days the first signs of corrosion appeared, and its development is visible mainly in the rebar area outside the concrete cube. The concrete cover effectively counteracts the development of the corrosion since only limited and local areas were noticed within the concrete interior. After 42 days of the corrosion acceleration experiment, corrosion of the rebar outside the concrete visibly increased and the corrosion within the concrete interior only slightly evolved. A similar conclusion might be drawn after 70 days of the experiment. Whereas rebar outside the concrete was heavily corroded, only local surface corrosion products appeared inside the concrete. It turned out that despite the accelerated corrosion environment, the concrete cover showed the protective ability of the concrete cover to counteract reinforcing steel corrosion mechanisms.

## 6. Pull-Out Test

**Figure 8:**
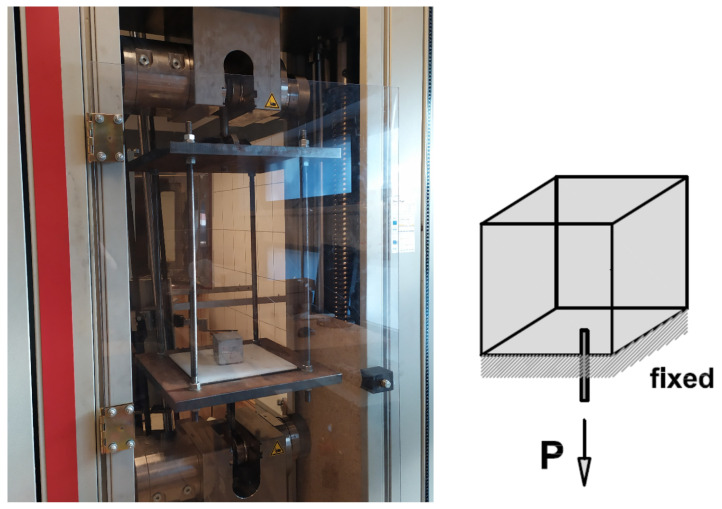
General view of the mechanical testing station.

**Figure 9:**
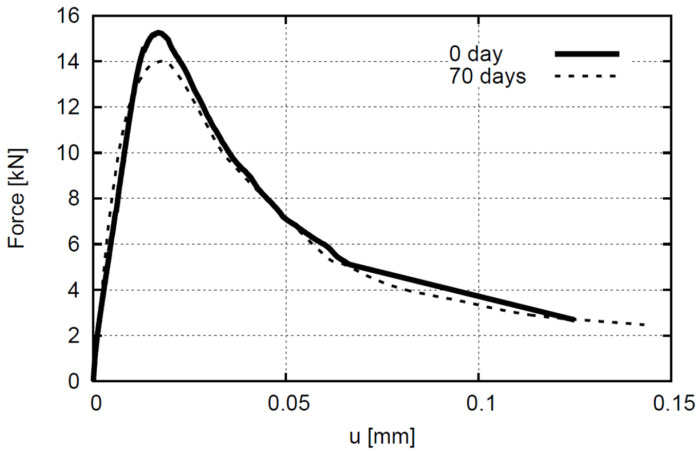
Experimental pull-out vertical force-displacement diagrams for reinforced concrete: without corrosion and after 70 days of accelerated corrosion test.

Pull-out quasi-static tests were carried out using the Zwick machine to observe if the steel corrosion mechanism negatively affects bond strength (Figure 8). The quasi-static tests were performed with a controlled displacement rate of 0.05 mm/min. Figure 9 presents vertical force (F) versus displacement curves (u) obtained for concrete reinforced steel subjected to the pull-out test. The maximum vertical force of concrete reinforced with steel bar without any corrosion was 15.21 kN (u = 0.16 mm), whereas concrete reinforced with steel bar after 70 days of the accelerated corrosion test was 14.05 kN (u = 0.17 mm). The difference in failure force was somewhat more connected with different material micro-structure than the local corrosion product inside the concrete. Tests were stopped before failure to allow samples to be scanned in one piece in micro-CT. However, after scanning, samples were again loaded to observe the failure mechanism. In both cases, failure took place due to concrete splitting.

**Figure 10:**
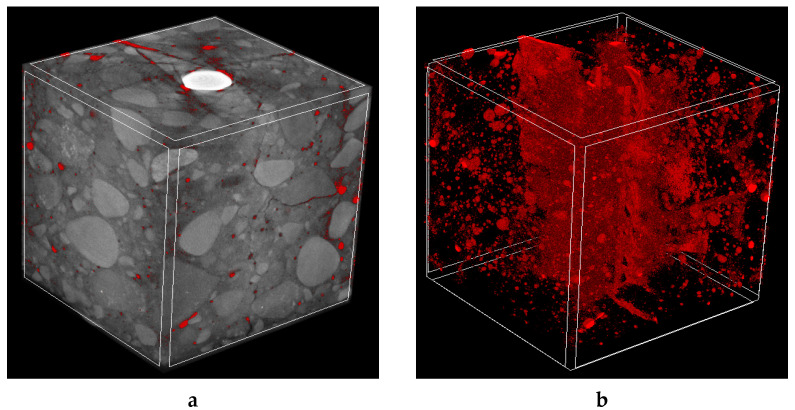
The 3D micro-CT images of cracked concrete cubes reinforced with steel bar after the pull-out test: (**a**) general view with air pores and cracks marked in red and (**b**) separated air pores and cracks.

**Figure 11:**
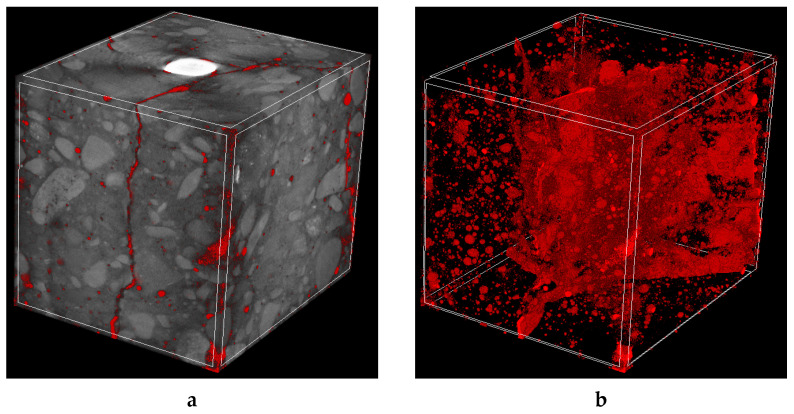
3D micro-CT images of cracked concrete cubes reinforced with steel bar after pull-out test: (**a**) general view with air pores and cracks marked in red and (**b**) separated air pores and cracks.

3D X-ray micro-CT investigations revealed that concrete cracking was similar for both cases (Figure 10 and Figure 11). The failure occurred due to the presence of radial cracks with a maximum width equal to approximately 0.25 mm.

## 7. Discussion

Corrosion of steel reinforcement is currently one of the most critical problems in preserving concrete structures. Corrosion of reinforcement leads to the weakening of the bond strength, resulting in deterioration of the structural integrity. As a result of corrosion, the initial cross-section of the reinforcement is reduced, which weakens the mechanical properties of the steel bar. Before chloride ions reach steel reinforcement, there is an initiation period. During this period, water and chloride ions reach the appropriate level necessary to start the corrosion process. This process can take more than 10 or 15 years until the destructive environmental influence reaches the reinforcement. Corrosion accelerated tests combined with X-ray micro-computed tomography (micro-CT) of concrete reinforced samples revealed that the chloride-accelerated corrosion test allows simulation and acceleration of the natural corrosion process. This technique can induce a high degree of corrosion within a controlled time.

The heterogeneity of tested material has a substantial impact on the local phenomena, such as the mechanism of the initiation, growth, and formation of fractures, which are responsible for the macroscopic behaviour of the material. Thus, concrete should be realistically described as a four-phase material composed of the cement matrix, aggregate particles, interfacial transition zones (ITZs), and macro voids assumed based on X-ray micro-CT images taken during experimental tests. Additionally, at this level of accuracy, the ribs of the steel bars should be modeled as well. The pull-out tests on uncorroded and corroded steel reinforcement concrete, allowed the material microstructure and cracking phenomenon to be observed using 3D X-ray micro-CT during the concrete splitting failure. The failure occurred due to the presence of radial cracks with a maximum width equal to approximately 0.25 mm.

Whereas rebar outside the concrete was heavily corroded due to the chloride-accelerated corrosion test, only local surface corrosion products appeared inside the concrete. It turned out that the concrete cover showed protective ability to counteract the reinforcing-steel corrosion mechanism despite the accelerated corrosion environment. Moreover, the bond strength between the reinforcement rebar and concrete was not affected since the failure force and failure mechanisms were similar for corroded and un-corroded samples.

## 8. Conclusions

Corrosion accelerated tests combined with X-ray micro-computed tomography (micro-CT) of concrete reinforced samples allow us to draw the following conclusions:

1. X-ray micro-CT allows visualization of material micro-structure, steel rebar with ribs, and air void distribution. Thus, the measured total air volume in the non-cracked reinforced concrete specimens ranged from 2.75% to 2.81%, whereas closed porosity varied from 2.39% to 2.49% and open porosity varied from 0.55% to 0.61%, respectively.
2. X-ray micro-CT scanning enables observation of the evolution of corrosion outside and inside the concrete sample after 14, 42, and 70 days. After 14 days of the chloride-accelerated corrosion test, noticeable corrosion changes within the rebar were noticed. Corrosion development is mainly visible in the rebar area outside the concrete cube. After 42 days of the chloride-acceleration experiment, corrosion of the rebar outside the concrete visibly increased while the corrosion within the concrete interior just slightly evolved. Similar conclusions might be drawn after 70 days of the experiment.

## 9. Future Perspectives

Our experiments will be continued with different steel corrosion ratios and various steel reinforcement diameters. X-ray micro-CT will be used to investigate the corrosion phenomenon and bond failure mechanism at the aggregate level. Furthermore, the experimental tests will be simulated using an isotropic coupled elastoplastic-damage constitutive model. It will be mainly for concrete enhanced by a characteristic length of microstructure in terms of integral non-local theory. The heterogeneity of tested material has a substantial impact on the local phenomena, such as the mechanism of the initiation, growth, and formation of fractures, which are responsible for the macroscopic behaviour of the material. Thus, concrete will be realistically described as a four-phase material composed of the cement matrix, aggregate particles, interfacial transition zones (ITZs), and macro voids assumed based on X-ray micro-CT images taken during experimental tests. Additionally, at this level of accuracy, the ribs of the steel bars will be modeled as well. The final goal is to introduce a new enhanced bond law that considers material heterogeneity.

## Figures and Tables

**Table 1:** Concrete recipe details.

Concrete Components	Concrete Mix (d_50_ = 2 mm, d_max_ = 16 mm)
Cement CEM II/A-LL 42.5 R	300 kg/m^3^
Sand (0–2 mm)	735 kg/m^3^
Gravel aggregate (2–8 mm)	430 kg/m^3^
Gravel aggregate (8–16 mm)	665 kg/m^3^
Superplasticizer	1.8 kg/m^3^
Water	150 kg/m^3^

**Table 2:** Properties of new concrete mix.

Concrete Mix	Temperature [°C]	Vebe SLUMP Test [mm]	Vebe Time [s]	Air Content [%]
Plain concrete mix (Table 1)	15.2	140	3.2	2.86

## Data Availability

Not applicable.

## Sample Availability

Not available.
